# Recombinant *Inga Laurina* Trypsin Inhibitor (ILTI) Production in *Komagataella Phaffii* Confirms Its Potential Anti-Biofilm Effect and Reveals an Anti-Tumoral Activity

**DOI:** 10.3390/microorganisms6020037

**Published:** 2018-04-28

**Authors:** Fábio C. Carneiro, Simone S. Weber, Osmar N. Silva, Ana Cristina Jacobowski, Marcelo H. S. Ramada, Maria L. R. Macedo, Octávio L. Franco, Nádia S. Parachin

**Affiliations:** 1 Grupo de Engenharia de Biocatalizadores, Instituto de Ciências Biológicas, Universidade de Brasília, CEP 70.790-900 Brasília-DF, Brazil; fbio.40@hotmail.com; 2 Pós-Graduação em Ciências Genômicas e Biotecnologia, Universidade Católica de Brasília, CEP 70.790-160 Brasília-DF, Brazil; marceloramada@gmail.com; 3 Faculdade de Ciências Farmacêuticas, Alimentos e Nutrição-UFMS, Laboratório de Purificação de Proteínas e suas Funções Biológicas-LPPFB, Cidade Universitária S/N-Caixa Postal 549, CEP 79.070-900 Campo Grande-MS, Brazil; weberblood@gmail.com (S.S.W.); bioplant@terra.com.br (A.C.J.); ligiamacedo18@gmail.com (M.L.R.M.); 4 Instituto de Ciências Exatas e Tecnologia, Universidade Federal do Amazonas, Itacoatiara, CEP 69100-000 Amazonas, Brazil; 5 S-Inova Biotech, Universidade Católica Dom Bosco, Programa de Pós-graduação em Biotecnologia, Campo Grande, CEP 79117-900 Mato Grosso do Sul, Brazil; osmar.silva@catolica.edu.br (O.N.S.); ocfranco@gmail.com (O.L.F.); 6 Centro de Análises Proteômicas e Bioquímicas. Programa de Pós-Graduação em Ciências Genômicas e Biotecnologia, Universidade Católica de Brasília, CEP 70.790-160 Brasília, Distrito Federal, Brazil; 7 Programa de Pós-graduação em Patologia Molecular, Universidade de Brasília, CEP 70.790-900 Brasília, Distrito Federal, Brazil

**Keywords:** *Komagataella phaffii*, heterologous expression, *Inga laurina* trypsin inhibitor (ILTI), biofilm assay, anti-tumor effect

## Abstract

Protease inhibitors have a broad biotechnological application ranging from medical drugs to anti-microbial agents. The *Inga laurina* trypsin inhibitor (ILTI) previously showed a great in vitro inhibitory effect under the adherence of *Staphylococcus* species, being a strong candidate for use as an anti-biofilm agent. Nevertheless, this is found in small quantities in its sources, which impairs its utilization at an industrial scale. Within this context, heterologous production using recombinant microorganisms is one of the best options to scale up the recombinant protein production. Thus, this work aimed at utilizing *Komagataella phaffii* to produce recombinant ILTI. For this, the vector pPIC9K+ILTI was constructed and inserted into the genome of the yeast *K. phaffii*, strain GS115. The protein expression was highest after 48 h using methanol 1%. A matrix-assisted laser desorption ionization–time-of-flight (MALDI–TOF) analysis was performed to confirm the production of the recombinant ILTI and its activity was investigated trough inhibitory assays using the synthetic substrate Nα-Benzoyl-D,L-arginine p-nitroanilide hydrochloride (BAPNA). Finally, recombinant ILTI (rILTI) was used in assays, showing that there was no significant difference between native and recombinant ILTI in its inhibitory activity in biofilm formation. Anti-tumor assay against Ehrlich ascites tumor (EAT) cells showed that rILTI has a potential anti-tumoral effect, showing the same effect as Melittin when incubated for 48 h in concentrations above 25 µg/mL. All together the results suggests broad applications for rILTI.

## 1. Introduction

Proteinase inhibitors (PIs) have an essential role in the development of several drugs, being used in the treatment of several diseases, such as cardiovascular disorders, treatment and prevention against cancer and HIV treatment by inhibiting the maturation of HIV virion [1,2]. Besides, PIs also play an essential role in agriculture, being used as bioinsecticides, anti-fungals and anti-bacterial agents [3,4]. Moreover, the global market for PIs was evaluated to be US$168 billion in 2017, and the global biopesticides market was worth US$3.3 billion in the same year [5,6]. Those molecules are naturally found in a wide variety of organisms, among which plants are known to produce a vast diversity of PIs that are located mainly in their reproductive and storage organs [7,8]. Those proteins are classified according to a class of proteinase in which the inhibitor acts against, which may be a serine, cysteine, aspartic and metalloprotease inhibitor [9]. The most studied and characterized PIs are those belonging to the families of the Kunitz-type inhibitors [10,11]. The primary mechanism of inhibition of a Kunitz-type protease inhibitor is by a non-covalent interaction with a serine protease, blocking the active site of the enzyme, forming an anti-parallel β-sheet between the protease and the inhibitor [12].

*Inga laurina* is a Brazillian tree belonging to Fabaceae family, sub-family Mimosoideae, which has an extensive geographical distribution, ranging from the north to the south of the country [13]. Previous studies reported that *I. laurina* seeds have a trypsin inhibitor, called ILTI (*Inga laurina* trypsin inhibitor) [14]. This molecule has 180 amino acids in its single polypeptide chain, a theoretical molecular mass of 19.8 kDa and 30–40% homology to Kunitz-family serine protease inhibitors [14]. ILTI revealed to have a high tolerance to pH variations, maintaining its residual activity close to 100% in a pH range from 2.0 to 10.0, and is extremely resistant to temperature variations, with its activity decreased only in temperatures above 70 °C [14]. 

Serine proteases, such as trypsin and chymotrypsin, are the main proteases presented in the mid-gut of insects, having an essential role in its development [15,16]. Previous studies showed that ILTI has an inhibitory effect in proteases extracted from the midgut of insects, reducing the tryptic residual activity by 95% in the mid-guts of *Sodoptera frugiperda* [17]. Besides, ILTI inhibited the growth and development of insects such as *Homalinotus coriaceus* and *Diatraea saccharalis*, where diets containing 0.25% and 0.1% of ILTI in the total protein cause 100% mortality and decrease its weight to 96%, respectively [8,17]. Therefore, ILTI has been shown to be a promising candidate to be used as a bioinsecticide.

The methylotrophic yeast *Komagataella phaffii*, widely known as *Pichia pastoris*, is commonly used as a recombinant protein-expression host. This yeast had the genus *Pichia* transferred to the genus *Komagataella*, due to phylogenetic analysis of its gene sequences. Therefore, in 2005, this yeast was reclassified as *Komagataella phaffii* [18,19,20]. Its advantages in recombinant protein production include its capacity of achieving high cell density and the presence of a strong, regulated, and inducible (by methanol) alcohol oxidase promoter (AOX1) [21,22]. This is the most frequently utilized promoter for recombinant protein production in this yeast [23,24,25,26]. Examples of PIs produced in *K. phaffii* under AOX1 promoter, includes the CmPI-II, and a Kunitz-type inhibitor ShPI-1A [27,28].

The advantage of using an inducible system for recombinant protein production is that optimization of cultivation conditions is done in two phases. Initially, it is necessary to achieve a high cell density using glucose or glycerol as a carbon source, and then initiate the phase of production, adding methanol to the culture as a secondary carbon source [29]. 

In this study, for the first time, we aimed to express a recombinant ILTI in the methylotrophic yeast *K. phaffii,* using a high cell-density fermentation. The protein expression was highest after 48 h using methanol 1%. A matrix-assisted laser desorption ionization–time-of-flight (MALDI–TOF) analysis was performed to confirm the production of the recombinant ILTI and its trypsin inhibitory activity was investigated through inhibitory assays using the synthetic substrate Nα-Benzoyl-D,L-arginine p-nitroanilide hydrochloride (BAPNA). Finally, recombinant ILTI was used in assays, showing that there was no significant difference between native and recombinant ILTI in its inhibitory activity in biofilm formation. Anti-tumor assay against Ehrlich ascites tumor (EAT) cells showed that recombinant ILTI (rILTI) has a potential anti-tumoral effect, showing the same effect as Melittin when incubated for 48 h in concentrations above 25 µg/mL. All together, the results suggests broad applications for rILTI. 

## 2. Materials and Methods

### 2.1. Strain Constructions and Culture Conditions

*Escherichia coli* strain DH10B was used as a host during the cloning procedures. It was propagated in a low-salt Luria-Bertani (LB) medium (0.5% yeast extract, 1% tryptone and 0.5% NaCl) at 37 °C. *K. phaffii,* strain GS115 (*his4-*), was used as an expression host for the recombinant ILTI (rILTI) and was cultured in YPD (1% yeast extract, 2% tryptone and 2% dextrose). The pPIC9K vector was obtained from Invitrogen, and the vector pPIC9K, harboring the *ILTI* gene, named pPIC9K+ILTI (Supplementary Figure S1), were synthesized by GenOne Biotechnologies (Rio de Janeiro, Brazil).

The recombinant vector pPIC9K+ILTI and the empty vector pPIC9K, used for control, was transformed into *E. coli* DH10B by the heat shock method as previously described [30]. Transformants were cultivated and selected on LB plates containing 100 µg/mL of ampicillin. Both vectors pPIC9K and pPIC9K+ILTI were linearized with the endonuclease SalI (Thermo Scientific, Waltham, MA, USA) and then inserted in *K. phaffii* GS115 by electroporation (Gene Pulser, Bio-Rad, CA, USA), according to the Easy Select *Pichia* Expression Kit instructions (Invitrogen, Carlsbad, CA, USA). Transformants were cultivated and selected by auxotrophic histidine selection on defined mineral medium plates [31], containing 1.8 g/L citric acid; 0.02 g/L CaCl_2_-2H_2_O; 12.6 g/L (NH_4_)_2_ HPO_4_; 0.5 g/L MgSO_4_-7H_2_O; 0.9 g/L KCl; 2.0 g/L agar and supplemented with 20 g/L glucose, 4.35 mL/L of trace salt stock solution (6.0 g/L CuSO_4_-5H_2_O; 0.08 g/L NaI; 3.0 g/L MnSO_4_-H_2_O; 0.2 g/L Na_2_MoO_4_-2H_2_O; 0.02 g/L H_3_BO_3_; 20.0 g/L ZnCl_2_; 65.0 g/L FeSO_4_-7H_2_O; 0.5 g/L CoCl_2_; 0.2 g/L biotin; 5.0 mL/L H_2_SO_4_) [31].

### 2.2. Heterologous Expression of Inga laurina Trypsin Inhibitor (ILTI) in K. Phaffii

After *K. phaffii* transformation, eight colonies of putative clones harboring the vector pPIC9K, named here as GS9K, and 30 colonies of putative clones harboring the recombinant vector pPIC9K+ILTI, named here as GSrILTI, were selected from defined mineral medium plates. Integration of the cassette into the *K. phaffii* genome was confirmed by colony polymerase chain reaction (PCR) using 5′AOX1 (5′-GACTGGTCCAATTGACAAGC-3′) and 3′AOX1 (5′-CGAAATGGCATTCTGACATGG-3′) primers according to Easy Select Pichia Expression Kit instructions (Invitrogen, USA).

One GSrILTI clone was selected, and one clone GS9K, to use it as a control, to verify the production of rILTI. The strains GS9K and GSrILTI were grown in 25 mL of defined mineral medium supplemented with 20 g/L glycerol and incubated at 28 °C for 24 h with rotary shaking at 200 rpm. After this period, the strains were transferred to 50 mL of defined mineral medium supplemented with glycerol 2% (*w/v*) with an initial OD_600_ of 0.1. The culture was grown under the same condition as described above until reaching an OD_600_ between 2 to 6. At that stage, the cell pellet was then harvested and resuspended in 50 mL of defined mineral medium, supplemented with methanol to a final concentration of 0.5% (*v/v*). Methanol 1% (*v/v)* was added to the culture every 24 h of growth. The cell-free supernatant was collected after 0, 12, 24, 36, 48, 60, 72 and 96 h of induction for monitoring protein production by sodium dodecyl sulfate polyacrylamide-gel electrophoresis (SDS–PAGE) [32]. 

### 2.3. High Cell-Density Fermentation of GSrILTI 

The clones GSrILTI and GS9K were pre-cultured in 100 mL defined mineral medium supplemented with glycerol 2% (*w/v*) medium for 48 h at 28 °C with rotary shaking at 200 rpm. The cells were then centrifuged at 1500 *g* and inoculated into the 3-L bioreactor (BioFlo 115, New Brunswick Scientific, Edison, NJ, USA) containing 1.5 L of defined mineral medium supplemented with glycerol 4% (w/v), for an initial OD_600_ of 0.5. The bioreactor was operated maintaining the temperature at 28 °C, pH at 5.5, controlled by automatic addition of 30% (*v/v*) ammonium hydroxide. The diluted oxygen (dO_2_) was maintained above 20% of saturation by controlling the stirring between 300 and 900 rpm and adjusting a constant air flow at 2 vvm. After depletion of glycerol, indicated by a sharp increase of dO_2_ (higher than 80%), the methanol adaptation phase was initiated by the addition of methanol 0.5 % (*v/v*) to the bioreactor. After the period of adaptation, indicated again, by a sharp increase of the dO_2_, the induction phase was initiated by adding 1% (*v/v*) methanol to the bioreactor. The induction period was maintained by 48 h with pulses of 1% (*v/v*) methanol always after the depletion of the methanol previously added. Samples were taken for determination of glycerol concentration and dry cell weight (DCW). 

### 2.4. Determination of Dry Cell Weight (DCW) and Substrate Concentrations 

Dry cell weight was determined by filtration of 5 to 10 mL culture broth at 0.22 µm membrane, washing the filtrate with 5 to 10 mL of 0.9% NaCl solution and subsequent drying at 180 °C for 24 h. OD_600_ of the culture broth was measured using a spectrophotometer, and the dry weight of the filters measured in an analytical balance. The correlation between DCW and OD_600_ was DCW (g/L) = 0.3121 × OD_600_, and showed a coefficient regression of R^2^ = 0.997.

Concentrations of glycerol were determined in cell-free samples by high-performance liquid chromatography (HPLC) (Prominence UFLC, Shimadzu, Kyoto, Japan) equipped with a Shim-Pack SCR-101H ion-exchange column (Shimadzu). The analysis was made in isocratic condition, with H_2_SO_4_ 5 mM as mobile phase, and a constant rate of 0.6 mL/min. Calibration was done by measuring standard points in the range of 10 to 0.03125 g/L glycerol.

### 2.5. Matrix-Assisted Laser Desorption Ionization–Time-of-Flight (MALDI-TOF) Analysis 

After 48 h of induction, the fermentation broth was centrifuged at 5000 g and the supernatant was collected. One milliliter of the supernatant was precipitate with trichloroacetic acid (TCA) 75% (*w/v*). After precipitation, 1 µL of the sample was mixed with α-cyano-4-hydroxycinnamic acid matrix (10 mg/mL, 50% (*v/v*) acetonitrile, 0.3% trifluoroacetic acid) in a 1:3 ratio and spotted in triplicate in a MTP 384 ground steel MALDI plate. Experiments were carried out in an Autoflex Speed MALDI-TOF/TOF (Bruker Daltonics, Billerica, Massachusetts, USA), controlled by FlexControl 3.0 software (Bruker Daltonics). Proteins average mass-to-charge ratio were obtained in positive linear mode over a range of *m/z* 9500–50,000 with external calibration using Protein Calibration Standard I (Bruker Daltonics). An in-source decay experiment was carried out using 2,5-dihydroxybenzoic acid (DHB) matrix (5 mg/mL, 30% (*v/v*) acetonitrile, 0.3% trifluoroacetic acid). Samples and matrix were directly applied in triplicate in a MTP 384 ground steel MALDI plate as follows: 1 µL of DHB matrix; 1 µL of sample after DHB crystallization; and 1 µL of DHB matrix after sample crystallization. In-source decay fragments were obtained in positive reflector mode over a range of *m/z* 1000–8000 with external calibration using 1 mg/mL bovine serum albumin (BSA) (Sigma-Aldrich, St. Louis, Missouri, USA). The software FlexAnalysis 3.0 (Bruker Daltonics) was used for mass spectrometric data analysis.

### 2.6. Trypsin Inhibitory Activity in Fermented Broth and Protein Quantification

The trypsin inhibitory activity of the fermentation broth after 48 h of induction was performed measuring the residual hydrolytic activity of trypsin towards the synthetic substrate BAPNA, as previously described [33]. One unit of trypsin activity was arbitrarily defined as the increase of 0.01 absorbance units at 410 nm. One inhibitor unit was defined as the amount of inhibitor that inhibited one unit of trypsin activity. The quantification of total protein in the fermentation broth, after 48 h of induction, was measured with the Bradford method [34], with BSA (Sigma-Aldrich) ranging from 0.781 to 500 µg/mL as standard. Experiments were performed in triplicates. 

### 2.7. Bacterial Strain Used to Biofilm Assay

Four gram-positive bacteria methicillin-susceptible *Staphylococcus aureus* MSSA ATCC80958, methicillin-resistant *Staphylococcus aureus* MRSA ATCC33591, *Staphylococcus epidermidis* ATCC 35984 and S. *epidermidis* ATCC 12228 (a non-slime production strain) were used in the biofilm assays. The *S. epidermidis,* known as slime-production INCQ 00650 ATCC 35984 (RP62A), was provided by the Coleção de Microrganismos de Referência em Vigilância Sanitária (CMRVS), FIOCRUZ-INCQS, Rio de Janeiro, RJ, Brazil. 

### 2.8. Biofilm Formation Assay and Determination of Minimal Biofilm Inhibitory Concentration (MBIC)

The four bacterial strains were grown in Mueller Hinton agar overnight at 36.5 °C, and a bacterial suspension in 0.9% NaCl, corresponding to 0.5 McFarland scale (1.5 × 10^8^ CFU/mL), was used. An in vitro microtiter plate-based model was performed as described for Staphylococci [35], with modifications. Briefly, 20 μL of each bacterial suspension equivalent to the McFarland 0.5 turbidity standard was inoculated onto 96-well microtiter plates with 170 μL of brain heart infusion (BHI) liquid medium. Subsequently, 10 µL of ILTI (previously purified from *Inga laurina* seeds) or GSrILTI fermented broth (rILTI) were added at different concentrations (range from 10 to 1000 µg/mL) to complete 200 μL of final volume per plate well, and the plate was incubated at 36.5 °C for 18 h. In positive controls, 10 μL of sterile distilled water was added instead of ILTI or rILTI. After incubation, the medium was removed, and the wells were washed three times with sterile distilled water. The remaining attached bacteria were fixed with 150 μL of methanol for 20 min. The adherent biofilm layer formed was stained with 0.5% crystal violet for 15 min at room temperature. Then, the dye was removed and this was followed by three washes with sterile distilled water. The preparations were then detained with 200 μL of 95% ethanol for 30 min. Finally, the optical density (OD) of the ethanol-dye suspension was measured at 450 nm. All the strains were tested in triplicate, and the average value for each sample was calculated. Values of higher than 100% represent a stimulation of biofilm formation in comparison with the positive control sample (untreated), in which the well was replaced by sterile distilled water. Vancomycin (20 µg/mL) was used as a negative control of bacteria growth and *S. epidermidis* (ATCC 12228) strain as negative control of biofilm formation. The minimal biofilm inhibitory concentration (MBIC) was defined as minimal concentration at which there were no observable adherent cells in wells stained with crystal violet, according to the approach described above. The concentrations of vancomycin in a range of 15 to 30 µg/mL (MBICs) to each isolates was used as the biofilm formation control.

### 2.9. Biofilm Detachment Assay and Determination of Minimal Biofilm Eradication Concentration (MBEC)

For preformed biofilm disassembly, the mature biofilm was first allowed to accumulate without any supplementation. Briefly, 20 μL of *S. epidermidis* (ATCC 35984) suspension was inoculated onto 96-well microtiter plates with 170 μL of BHI liquid medium at 36.5 °C for 24 h. The ILTI and rILTI influence under biofilm disassembly were pre-established with 24 h old biofilms; then, after this period, biofilms were treated with a range of each ILTI or GSrILTI fermentation broth, containing the recombinant ILTI, concentrations (10 to 100 µg/mL) and incubated at 36.5 °C for an additional 18 h. The amount of residual biofilm was measured using the CV assay, as described above. 

The minimal biofilm eradication concentration (MBEC) was evaluated using a modified version of the Calgary biofilm device method previously described [36]. Briefly, biofilms were initially formed onto 96-well microtiter plates and then wells were washed carefully with 0.9% NaCl to remove planktonic bacteria. The biofilms that remained in the wells were exposed to a range of each ILTI or GSrILTI fermented broth concentrations (10 to 100 µg/mL) to 200 μL final volume of BHI liquid medium and then incubated for 24 h at 36.5 °C. After that, wells were washed carefully to remove residual molecules and fresh BHI liquid medium was placed in each well. After 24 h at 36.5 °C, the biofilm with the bacteria that survived the treatment was left to grow in the absence of them and produce detectable turbidity (defined as bacterial recovery) at OD_600_. The MBEC value is the lowest molecule concentration that prevented the regrowth of bacteria from the treated biofilm. Each MBEC experiment at each ILTI or rILTI concentration was repeated in triplicate for each isolate tested. Non-biofilm forming isolate *S. epidermidis* (ATCC 12228) was used as negative control, and wells containing the standard established drug (vancomycin) were used as positive control.

### 2.10. Ethics Statement

The use of mice was conducted following the regulations set forward by the respective national animal protection committees and following European Community Directive 86/609 and the U.S. Association for Laboratory Animal Care recommendations for the care and use of laboratory animals. All the techniques/procedures have been refined to provide for maximum comfort/minimal stress to the animals. The performed experiments have been approved by Animal Ethics Committees of the Universidade Catolica Dom Bosco (AECs/UCDB), number 005/2016.

### 2.11. Anti-Tumor Activity Evaluation

Ehrlich ascites tumor (EAT) cells were provided as a courtesy sample by Department of Pathology of UNESP, Botucatu-SP, Brazil. Cells were maintained in vivo in ascites form by successive transplantation of 6 × 10^6^ cells/mice in a volume of 0.2 mL in phosphate buffered saline (PBS) [37]. Seven days after the inoculation of EAT cells in the abdominal cavity of the mice, cells were harvested by needle aspiration, washed with PBS [38]. Cells were cultured in Roswell Park Memorial Institute medium (RPMI) 1640 supplemented with HEPES (25 mM), L-glutamine (2 mM), sodium bicarbonate (25 mM), 10% fetal bovine serum (FBS) antibiotics (100 U/mL penicillin and 100 μg/mL streptomycin) at 37 °C in 5% in CO_2_ incubator. Cell viability was determined by the trypan blue dye exclusion test [37].

Cells were seeded in 96-well microtiter plates in a concentration of 2.0 × 10^5^ cells per well, in RPMI medium, supplemented with different concentrations of final GS9K fermentation broth, used as negative control, or with different concentrations of final GSrILIT fermentation broth (3.125 to 400 μg/mL). After 24 h of the incubation, a 3-[4,5-dimethylthiazol-2-yl\-2,5-diphenyl-tetrazolium bromide (MTT) protocol was performed. Briefly, 60% of the medium was removed, and 0.1 mg mL−1 of MTT was added to each well, and the plate was incubated for 4 h, in 5% CO2, at 37 °C in the dark. The blue formazan product generated was dissolved by the addition of 100 μL of DMSO (Mallinckrodt, Staines-upon-Thames, England) per well. Plates were then gently swirled for 5 min, at room temperature, to dissolve the precipitate. Absorbance was monitored at 575 nm using a microplate spectrophotometer (Bio-Tek, Winooski, VT, USA). Viability was determined as a percentage of the maximum value after subtracting the background. Results were expressed as the percentage of each sample compared to the negative control.

### 2.12. Statistical Analysis

Values are expressed as means of at least three separate determinations ± standard error of the mean (SEM). The statistical analysis was carried out by analysis of variance (ANOVA), followed by Tukey’s test (GraphPad Prism 5, GraphPad Software, San Diego, CA, USA). *p*-values of less than 0.05 were considered as statistically significant (* *p* < 0.05, ** *p* < 0.01 and *** *p* < 0.001). 

## 3. Results and Discussion

### 3.1. Expression of Recombinant ILTI (rILTI)

**Figure 1:**
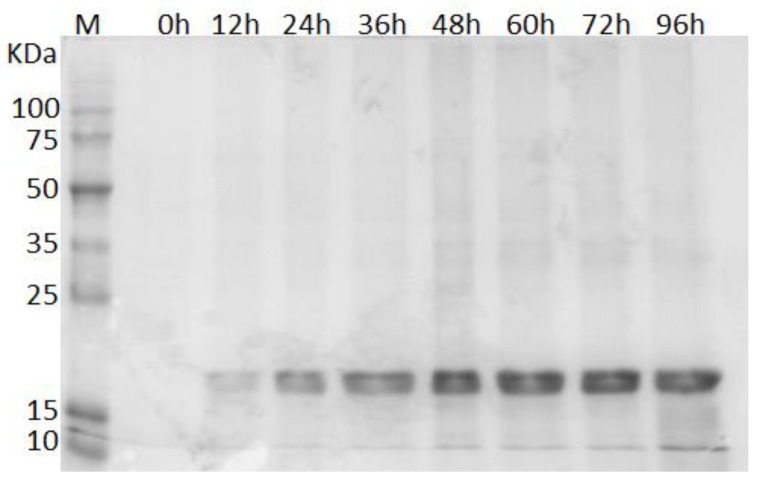
Sodium dodecyl sulfate polyacrylamide–gel electrophoresis (SDS–PAGE) (12%) of the samples collected during the 96 h induction. Lane M contains the broad range protein molecular weight markers (Promega); at its side it is possible to verify the molecular weight of each protein marker. Each of the following lanes have the culture supernatant where the time of induction is indicated above.

In this study, for the first time, the protein ILTI was successfully produced and secreted in K. phaffii GS115. As can be observed in Figure 1, the recombinant protein was produced during the induction of the GSrILTI strain, where a gradual increase of a band of approximately 20 kDa can be observed. It was possible to observe the production of the recombinant protein after 12 h of induction with methanol, whereas for the control strain GS9K no protein was secreted during its induction, as can be seen in Figure S2. The apparent size of the secreted protein was in agreement with its theoretical molecular weight of 19.8 kDa. Apparently, no difference in the expression of the inhibitor was observed after 48 h of induction (Figure 1). 

It is possible to state, based on Figure 1, that rILTI was basically the only observable secreted protein after methanol induction, and that the rILTI observed molecular weight matches the native ILTI [14]. Nevertheless, SDS–PAGE analysis is not the only method used to check recombinant protein production. Direct inhibition assay of the fermented broth to verify if the recombinant PI was produced, by monitoring the reduction in trypsin residual activity are also used [27,39]. In addition, authors also perform mass spectrometry analyses by MALDI–TOF to confirm the production of the recombinant inhibitor [27,39,40] by checking the intact PI mass or by determining the amino acid sequence of protein fragments [27,39,41].

### 3.2. Production of rILTI in the Bioreactor

**Figure 2:**
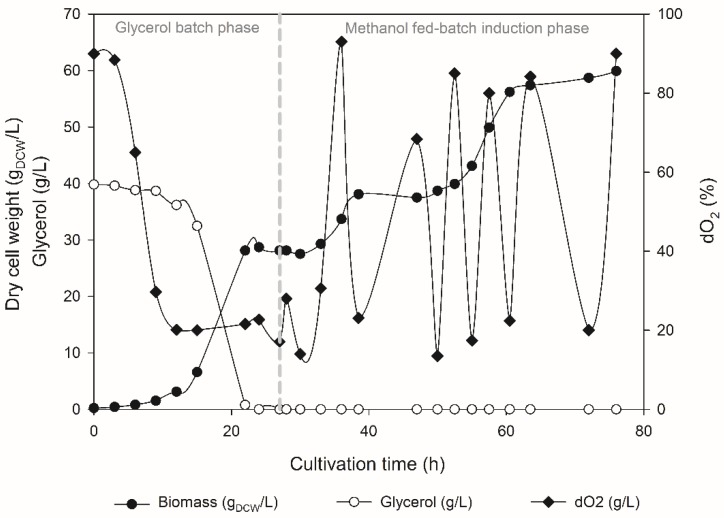
Fermentation profile of the GSrILTI strain, indicating the two different batch phases during the cultivation.

Since it was possible to confirm that the rILTI was being produced, the GSrILTI strain was transferred to the bioreactor with the aim of quantifying the production of rILTI and obtaining the initial kinetic parameters (Figure 2). For that, fed-batch fermentation was undertaken, where initially a batch-phase cultivation was conducted using glycerol as a prior carbon source. Both substrates, glycerol and glucose, enable *K. phaffii* to reach high-density cultivation at higher rates when compared to other carbon sources such as methanol [42]. However, it is more suitable to use glycerol as a prior carbon source in a methanol-fed batch fermentation, since its use decreases the time of transition phase to methanol when compared to glucose [43]. Later, when 28 g/L of biomass was achieved, a transition phase was conducted followed by the induction phase with pulses of methanol. The growth profile of the recombinant strain is shown in Figure 2.

The pulse-strategy of alimentation/induction is not widely used for protein production in bioreactors. However, this strategy provides a fast approach to determine the specific parameters of the strain, which is crucial to optimizing fed-batch cultivation [44]. For instance, using the data obtained in Figure 2 it was possible to establish a maximum growth rate in glycerol and methanol, 0.22 h^−1^ and 0.044 h^−1^, respectively, which can be used to further develop a fed-batch strategy to optimize rILTI production. Indeed fed-batch cultivation was carried out, with an automated continuous feeding rate of methanol. However, with this setup, an accumulation of methanol was observed during the feeding phase, resulting in cell death. Therefore, future experiments are necessary to optimize fed-batch cultivation of the GSrILTI strain. For this, it would be necessary to carry out a meticulous control of the addition of methanol to the bioreactor, and, as is observed in other studies, for the production of PIs in *K. phaffii* using P_AOX1_, control the feed of methanol leaving it to basal levels [27,28].

### 3.3. Confirmation of rILTI Production by MALDI–TOF Analysis

**Figure 3:**
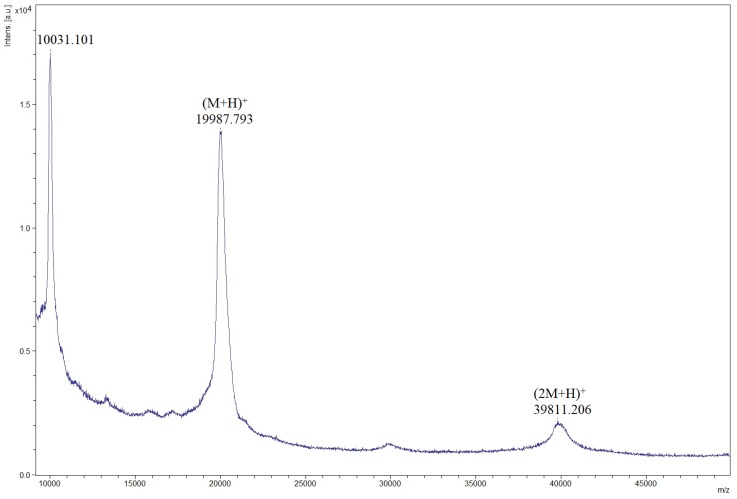
Mass spectrometry spectrum obtained from the final fermentation precipitate of GSrILTI strain. The *m/z* 19,987.793 ion corresponds to the recombinant ILTI (19.8 kDa), and the *m/z* 39,811.206 ion corresponds to a single charged dimer of the recombinant ILTI.

MALDI–TOF analysis is always associated with other previous analyses to detect or confirm the recombinant PI [27,39,40]. Therefore, in this study a mass spectrometry analysis of intact proteins was performed in order to confirm if the rILTI was, indeed, produced. As can be seen in Figure 3, an ion of *m/z* 19987 was obtained in the spectrum of the fermentation broth after 48 h of induction, which cannot be seen in the spectrum of the GS9K control strain (Figure S3), indicating that this ion is the rILTI. The ion *m/z* 39811 possibly corresponds to a single charged dimer of the PI, as Kunitz protease inhibitors are known to form multimers in solution [45,46]. The results herein confirms the SDS–PAGE analysis (Figure 1 and Figure S2).

### 3.4. Determination of rILTI Activity

As shown in Table 1, the inhibitory activity could be confirmed in the final fermentation sample of the GSrILTI strain. Moreover, Table 1 demonstrates that the supernatant of the strain harboring the empty plasmid, strain GS9K, has an inhibitory activity that can be disregarded.

As it can be seen, the recombinant ILTI produced by GSrILTI strain was shown to have potent inhibitory activity against trypsin. When comparing the production of this inhibitor with the production of the CmPI-II protease inhibitor previously reported [27], it can be seen that rILTI specific activity was similar to that previously observed (2.62 U/mg). However, due to the lack of studies in which the production of a trypsin inhibitor in *K. phaffii* was performed, it is difficult to compare the particular activity obtained here with that achieved in the literature. Moreover, comparing the results obtained here with those obtained in other studies is only possible if both used the same parameters to measure both the amount of total protein and the number of inhibitor units. Therefore, if we take into account the specific activity of 1285 U/mg of that previously obtained [28], we cannot assume that the specific activity achieved here is not significant, since the parameters used in the previous study to determine the inhibitory activity in the final fermented broth is different from that used here.

Since no control of methanol feed was made into the bioreactor, it is believed that by controlling the feed profile it is possible to increase even further the production of rILTI and, consequently, increase its specific activity. Therefore, the data obtained here is an initial step to optimize fed-batch cultivation of GSrILTI.

### 3.5. Anti-Biofilm Activity of rILTI

Biofilm infection represent a serious health threat worldwide today mostly due to the appearance of antibiotic-resistant strains. The ILTI was first isolated from *Inga laurina* and characterized as a Kunitz trypsin inhibitor [14] after a primary screening, demonstrating potent inhibition of the proliferation of *Candida tropicalis* and *Candida buinensis*, but with no effect on planktonic bacterial cell proliferation [47]. Structure–activity relationship studies have confirmed no significant overlap between anti-biofilm and anti-microbial (versus planktonic bacteria) activities [48]. Indeed, while ILTI did not present anti-microbial activity, in the present study our results have highlighted an interesting inhibitory activity on biofilm formation and a capability to disrupt the pre-established biofilm. Table 2 shows that there was no significant difference between ILTI and GSrILTI fermented broth (rILTI) MBIC (*p*-value = 0.108) and/or MBEC (*p*-value = 0.378) values, indicating that the heterologous expression technique used here to produce the rILTI did not compromise the biological activity of this trypsin inhibitor.

Interestingly, there were no significant differences in the MBIC values of rILTI between the methicillin-susceptible (*S. aureus* MSSA ATCC80958) and methicillin-resistant (*S. aureus* MRSA ATCC33591, *S. epidermidis* ATCC 35984 and S. *epidermidis* ATCC 12228) strains (*p*-value = 0.418), suggesting a broad-spectrum performance. Furthermore, we highlighted the promising ability of rILTI to efficiently eradicate the established 24 h-old biofilm, with the MBEC range value from 120 to 220 µg/mL (Table 2). During the biofilm detachment process, clusters of bacteria or single bacteria are reported to be expelled from the biofilm; this process can be stimulated by pulmonary surfactant monolayers (PSM) and by enzymes that degrade biofilm matrix molecules, such as nucleases and proteases. The relevance of the latter mechanism for infection is unclear [49]. Therefore, the exact roles of degradative enzymes in staphylococcal biofilm structuring and dissemination/dispersal need to be delineated. We are unable to speculate on the mechanism by which ILTI induces biofilm dispersal; however, our findings open up opportunities for future studies in this area.

### 3.6. Evaluation of Anti-Tumoral Activity

**Figure 4:**
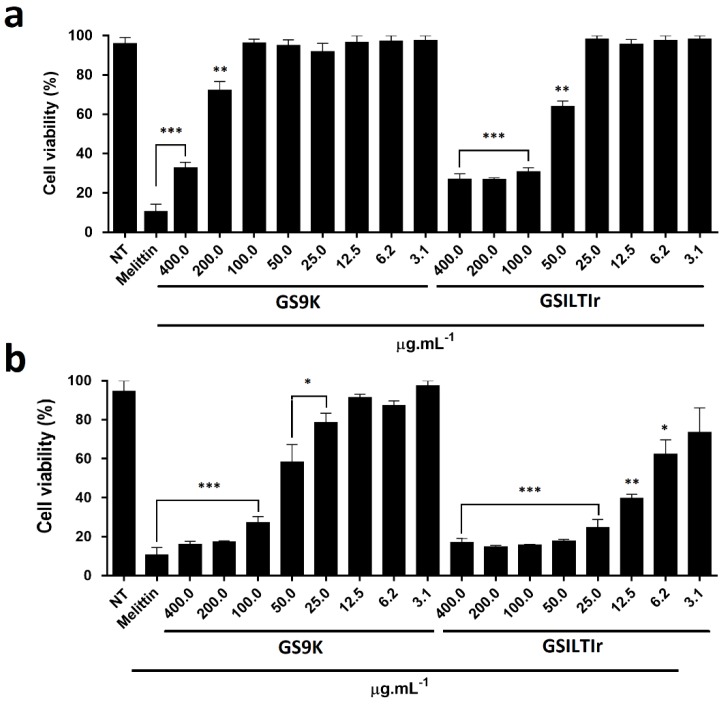
Anti-tumor effect of both final fermented broth against Ehrlich’s ascites tumor (EAT) cells. Viability effect, after 24 (**a**) and 48 h (**b**) incubation time of GS9K fermented broth and GSrILTI fermented broth, at different concentrations. Melittin used as positive control at 25 µg/mL. * *p* < 0.05, ** *p* < 0.01 and *** *p* < 0.001 (analysis of variance (ANOVA), post doc Bonferroni).

The anti-tumor effect of PIs has already been demonstrated in different cell lines. It was shown by Bezerra et al. [50], that a Kunitz-type trypsin inhibitor extracted from the seeds of *Inga vera* (IVTI) has an anti-proliferative effect on the CACO-2 cell line at 200 µg/mL. Additionally, Fang et al. [51] isolated a Kunitz-type trypsin inhibitor from Korean large black soybeans (KBTI), which weakly inhibited the proliferation of NPC, CNE-2, HNE-2, MCF-7 and HepG2 cells. Therefore in order to investigate the potential anti-tumoral activity of rILTI, EAT cells were utilized using the GSrILTI fermentation broth containing rILTI, the fermentation broth of GSK9 strain (negative control) and Melititin (positive control) (Figure 4).

The final fermented broth of the GS9K control strain showed to have an cytotoxicity effect for concentrations above 200 µg/mL, reducing the cell viability about 25% (*p* < 0.01) after 24 h of incubation (Figure 4a). When the same assay was performed using the fermented GSrILTI broth, the cytotoxic effect was detected in the concentrations of 50 and 12.5 µg/mL at 24 and 48 h of incubation (Figure 4). Statistically, GS9K fermented broth only showed the same anti-tumor effect as the positive control (Melittin at 25 µg/mL) in concentrations above 100 µg/mL when incubated for 48 h, and at 400 µg/mL when incubated for 24 h (Figure 4). In contrast, GSrILTI fermented broth, statistically, had the same anti-tumor effect of Melittin control in concentrations above 100 µg/mL, when incubated for 24 h. When incubated for 48 h the same anti-tumor effect as Melittin control was observed in levels above 25 µg/mL (*p* < 0.001).

The IC50 represents the concentration of inhibitor required to inhibit 50% of cell viability, and was determined after 24 h and 48 h of incubation for EAT cells (Figure S4). The IC50 values of 50.118 µg/mL (1.707 log µM) and 7.585 µg/mL (0.903 log µM) were obtained for GSILTIr fermented broth after 24 h and 48 h of incubation, respectively (Figure S4). Meanwhile, the IC50 values of 207.491 µg/mL (2.317 log µM) and 67.608 µg/mL (1.729 log µM) were obtained for negative control, GS9K-fermented broth (Supplementary files S4). However, GSrILTI final fermented broth was shown to have a higher significant impact, since its IC50 values are about four times lower at 24 h of assay and almost 9 times lower at 48 h of assay than those obtained from the negative control. Consequently, it can be said, that rILTI has a potential anti-tumor effect, and so it is of interest, in future experiments, to investigate the anti-tumor effect of the purified molecule and even perform in vivo assays in order to test its use as a possible pharmaceutical drug.

## 4. Conclusions

Here, for the first time, this protein was successfully produced in *K. phaffii* in a high cell-density fermentation, with methanol addition by pulse. The broad biotechnological applicability of the recombinant rILTI has been demonstrated by the confirmation of its anti-biofilm activity and its newly found anti-tumoral potential. Altogether, the results presented here are an initial step for development of innovative bio-based products using rILTI.

## Figures and Tables

**Table 1:** Amount of protein and trypsin inhibitor produced by GS9K and GSrILTI strains at the end of each fermentation.

Strain	Protein (µg/mL)	Inhibitory Activity (U)	Specific Activity (U/mg)
**GS9K**	64,571	30,53	0,18
**GSrILTI**	146,984	351,27	2,07

**Table 2:** The minimal biofilm inhibitory concentration (MBIC) and minimal biofilm eradication concentration (MBEC) (µg/mL) values of ILTI, rILTI, and Vancomycin in *Staphylococcus* species. not applicable (NA); not performed (-).

Strains	MBIC (µg/mL)			MBEC (µg/mL) Range		
	ILTI	rILTI	Vancomycin	ILTI	rILTI	Vancomycin
***S. epidermidis*** **(ATCC 35984)**	25	25	15	150–200	120–220	80–100
***S. epidermidis*** **(ATCC 12228)**	NA	NA	NA	NA	NA	NA
***S. aureus*** **MSSA ATCC80958**	20	20	25	⁻	⁻	80–120
***S. aureus*** **MRSA (ATCC33591)**	25	30	30	⁻	⁻	100–250

## Supplementary Materials

Supplementary materials can be found at http://www.mdpi.com/2076-2607/6/2/37/s1.
