# Supplementary material for: Recombinant Inga Laurina Trypsin Inhibitor (ILTI) Production in Komagataella Phaffii Confirms Its Potential Anti-Biofilm Effect and Reveals an Anti-Tumoral Activity
Source: Microorganisms. 2018 Apr 28;6(2):37. doi: 10.3390/microorganisms6020037 (PMC6027459; doi:10.3390/microorganisms6020037)
Supplement: Supplementary file 1 [file microorganisms-06-00037-s001.pdf]

Microorganisms

Recombinant *Inga Laurina* Trypsin Inhibitor (ILTI) Production in *Komagataella Phaffii* Confirms its Potential Anti-Biofilm Effect and Reveals an Anti-Tumoral Activity

Fábio C. Carneiro [1,2], Simone S. Weber [3,4], Osmar N. Silva [5,6,7], Ana Cristina Jacobowski [3], Marcelo H. S. Ramada [2], Maria L. R. Macedo [3], Octávio L. Franco [5,6,7] and Nádia S. Parachin [1,2,*]

[1] Grupo de Engenharia de Biocatalizadores, Instituto de Ciências Biológicas, Universidade de Brasília, CEP 70.790-900 Brasília-DF, Brazil; fbio.40@hotmail.com

[2] Pós-Graduação em Ciências Genômicas e Biotecnologia, Universidade Católica de Brasília, CEP 70.790-160 Brasília-DF, Brazil; marceloramada@gmail.com

[3] Faculdade de Ciências Farmacêuticas, Alimentos e Nutrição-UFMS, Laboratório de Purificação de Proteínas e suas Funções Biológicas-LPPFB, Cidade Universitária S/N-Caixa Postal 549, CEP 79.070-900 Campo Grande-MS, Brazil; weberblood@gmail.com (S.S.W.); bioplant@terra.com.br (A.C.J.); ligiamacedo18@gmail.com (M.L.R.M.)

[4] Instituto de Ciências Exatas e Tecnologia, Universidade Federal do Amazonas, Itacoatiara, CEP 69100-000 Amazonas, Brazil

[5] S-Inova Biotech, Universidade Católica Dom Bosco, Programa de Pós-graduação em Biotecnologia, Campo Grande, CEP 79117-900 Mato Grosso do Sul, Brazil; osmar.silva@catolica.edu.br (O.N.S.); ocfranco@gmail.com (O.L.F.)

[6] Centro de Análises Proteômicas e Bioquímicas. Programa de Pós-Graduação em Ciências Genômicas e Biotecnologia, Universidade Católica de Brasília, CEP 70.790-160 Brasília, Distrito Federal, Brazil

[7] Programa de Pós-graduação em Patologia Molecular, Universidade de Brasília, CEP 70.790-900 Brasília, Distrito Federal, Brazil

* Correspondence: nadiasp@unb.br or nadiasp@gmail.com

Supplementary Materials:

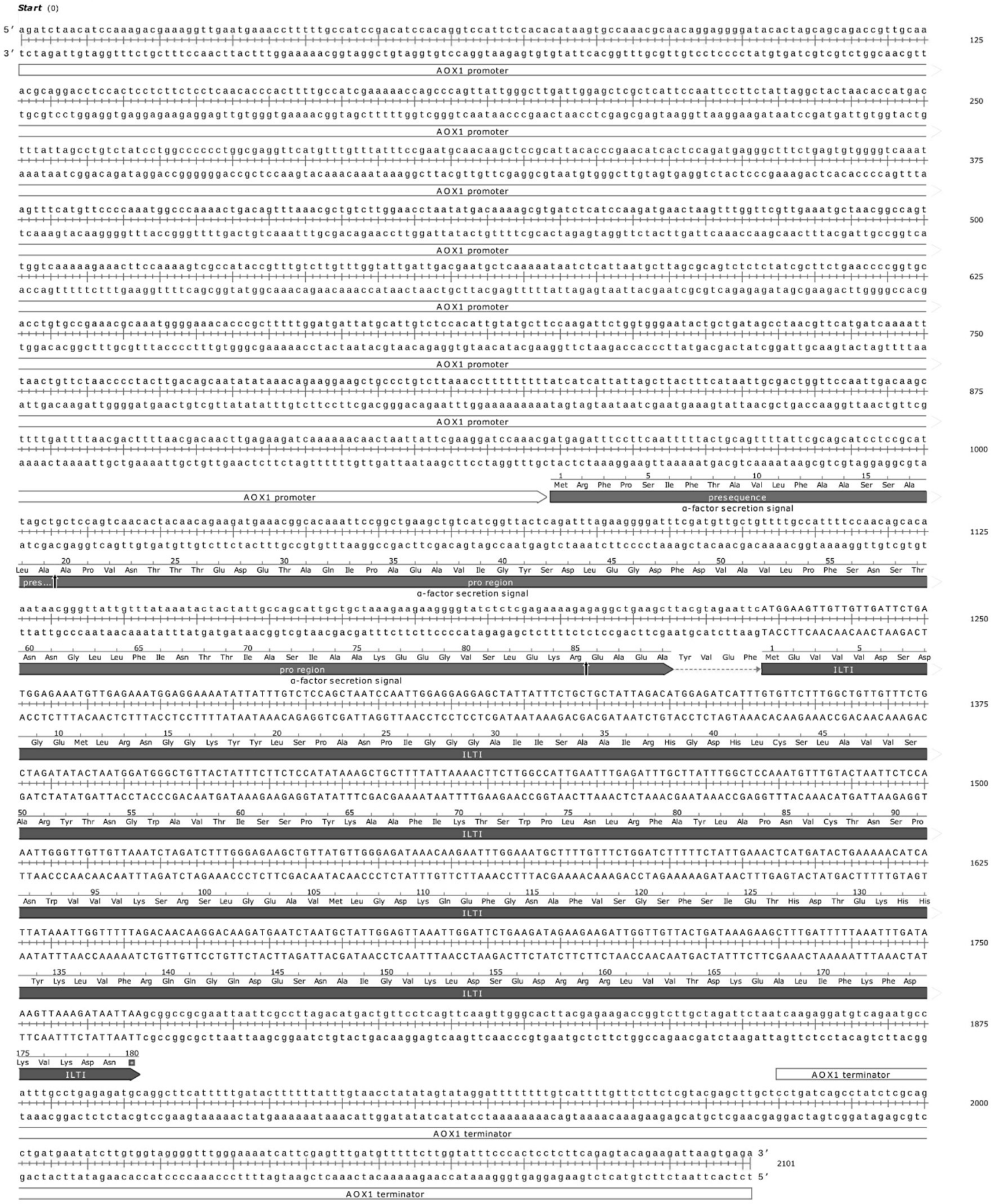

Figure S1. Nucleotide sequence of pPIC9K+ILTI expression cassette. In the figure it can be observed the sequence of AOX1 promoter region, followed by the α-factor secretion signal, showing the place of Kex2 cleavage sites by an arrow. The gene sequence can be observed right after the last Kex2 cleavage site, and was designed from native ILTI amino acid sequence (show in the figure), optimized for *K. phaffii* expression taking into account the published yeast codon usage. Lastly in can be observed the AOX1 terminator region.

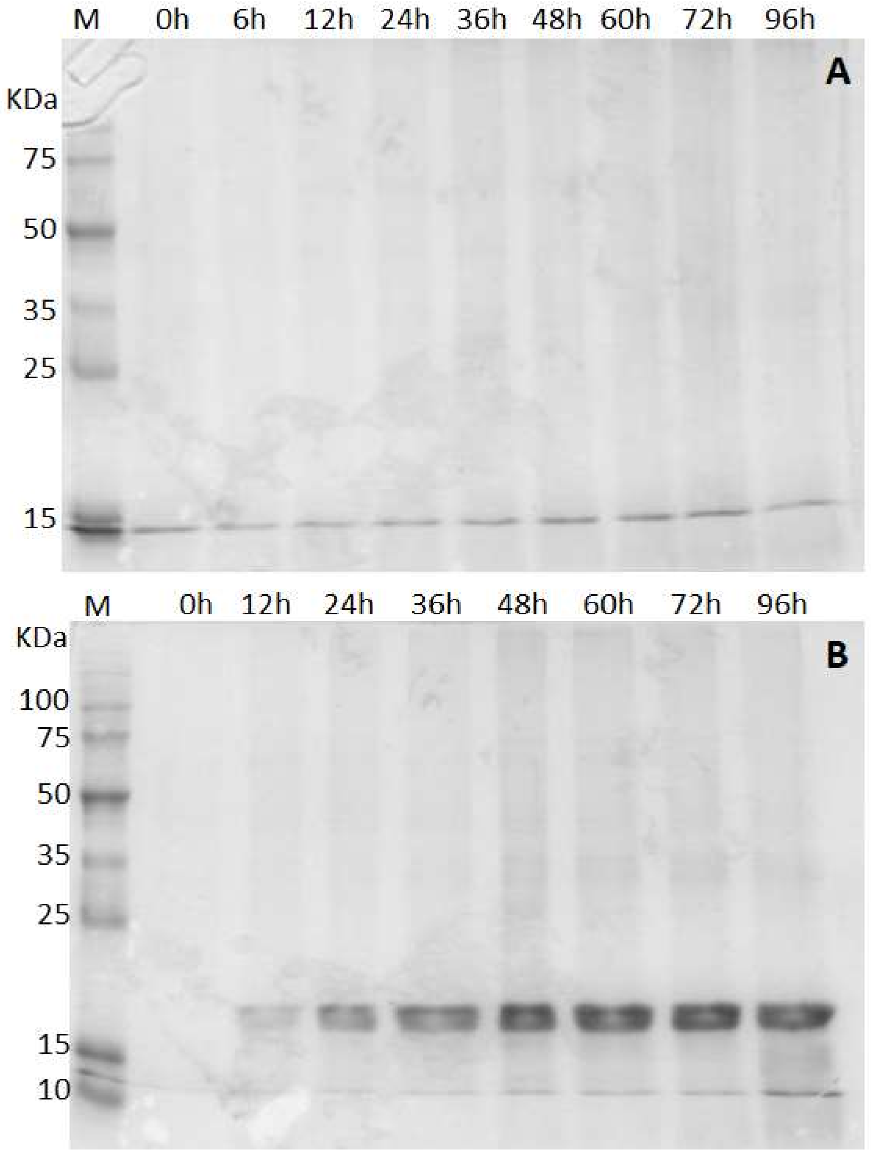

Figure S2. SDS-PAGE (12%) of cell-free samples collected during the 96 hours induction. Lane M contains the Broad Range Protein Molecular Weight Markers (Promega), at its side, it is possible to verify the molecular weight of each protein marker. In each of the followed lanes have the culture supernatant where the time of induction is indicated above. A) Secretory profile of the samples collected during the induction of GS9K strain; B) Secretory profile of the samples collected during the induction of GSILTIr strain.

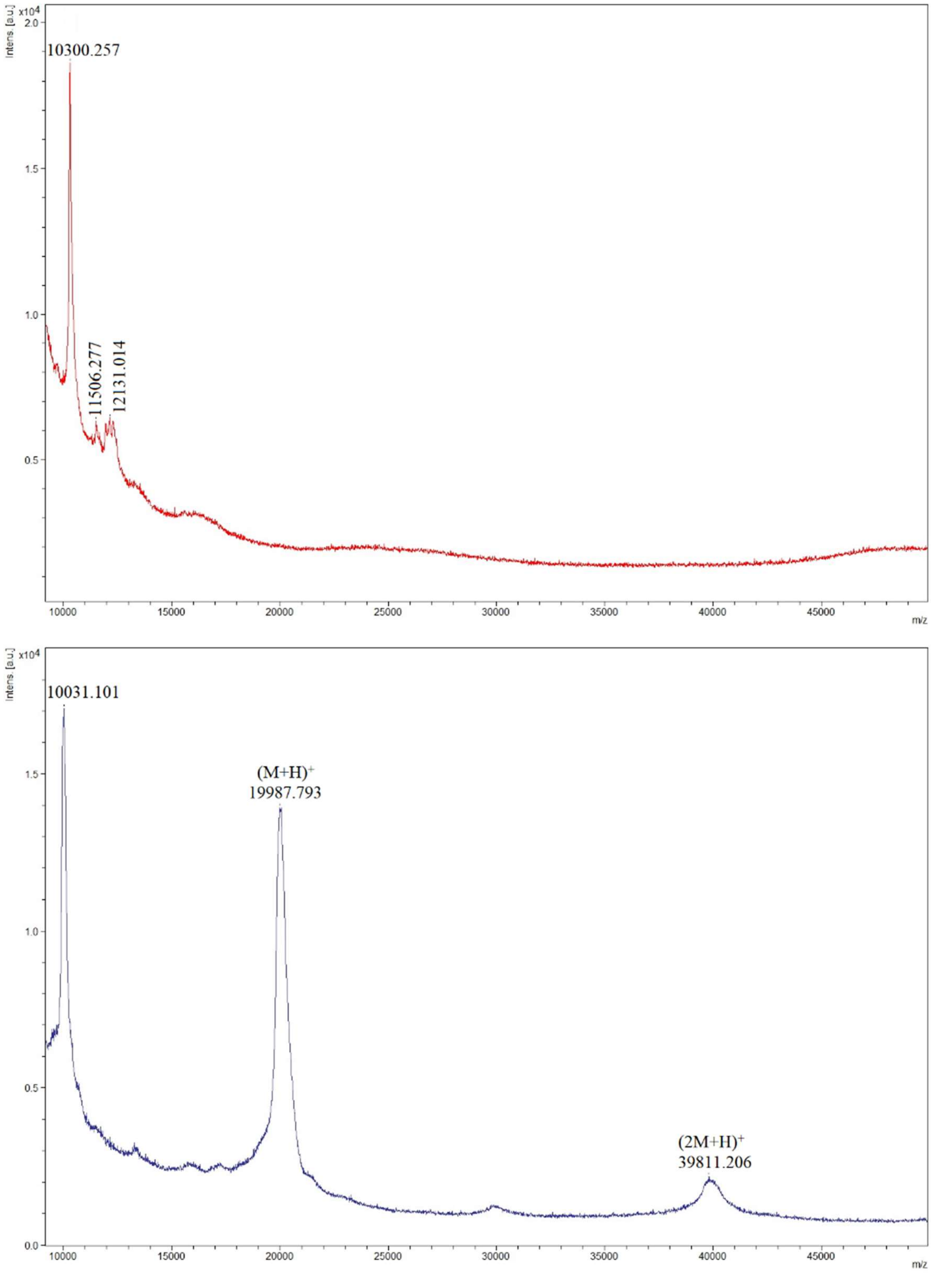

Figure S3. A) Spectrum obtained from the final fermentation precipitate of GS9K strain. The m/z correlation of each ion is indicated above itself; B) Spectrum obtained from the final fermentation precipitate of GSILTIr strain. The 19987.793 m/z ion corresponding to the recombinant ILTI (19.8 kDa), and the 39811.206 m/z ion corresponding to a single charged dimer of the recombinant ILTI.

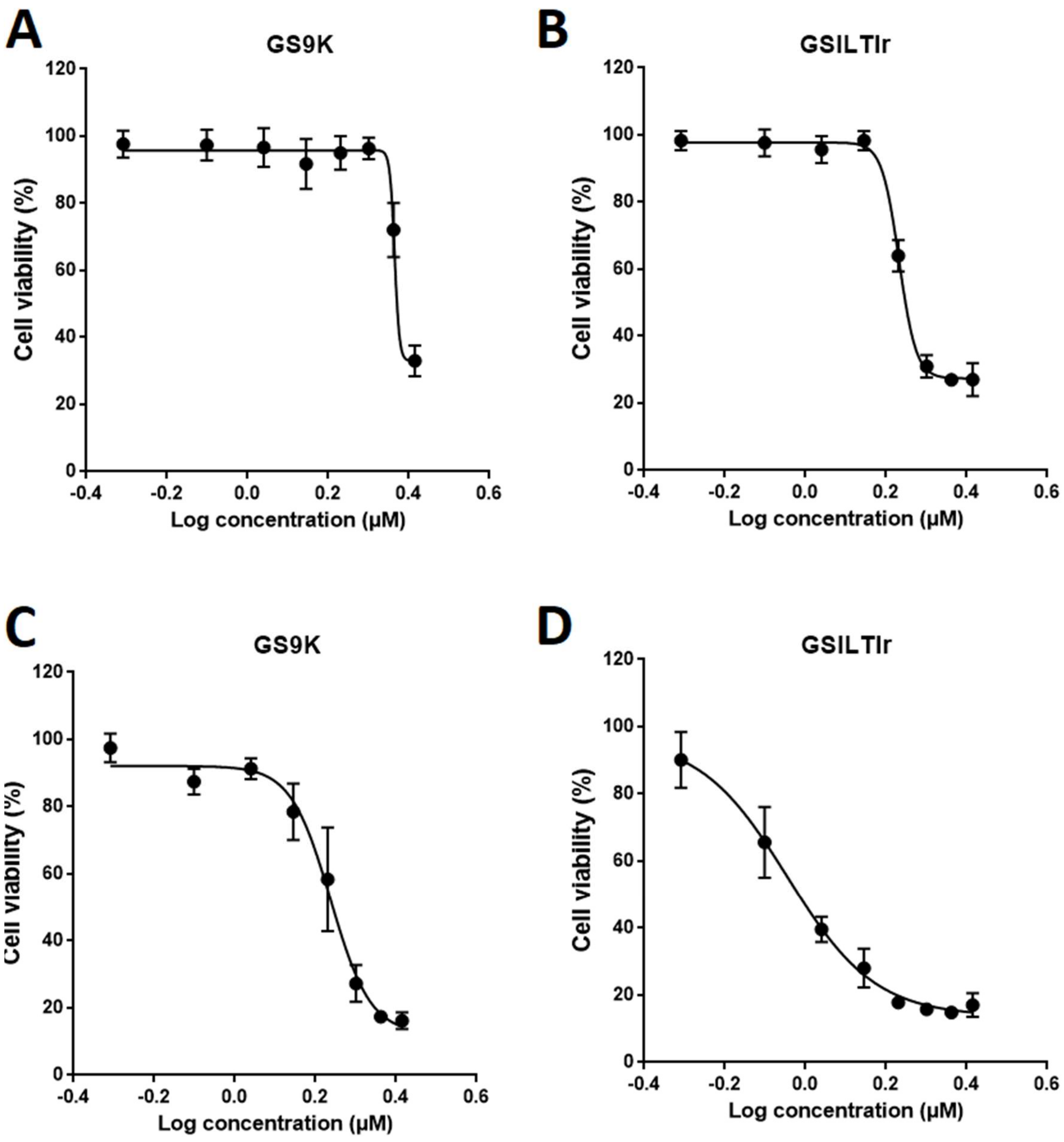

Figure S4. Determination of IC50 values of GSILTIr and GS9K final fermented broth against EAT cells after 24 (A and B) and 48 hours (C and D) of incubation. The graphic shows the fit of cell viability (%) vs a log concentration of inhibitor (μM) to IC50 value for the cell viability assay, implemented on GraphPad Prism v6.0 software.
